# Robotic-assisted surgery for gynecological indications in children and adolescents: European multicenter report

**DOI:** 10.1007/s11701-023-01767-9

**Published:** 2024-01-13

**Authors:** Ciro Esposito, Thomas Blanc, Claudia Di Mento, Quentin Ballouhey, Laurent Fourcade, Mario Mendoza-Sagaon, Annalisa Chiodi, Roberto Cardone, Maria Escolino

**Affiliations:** 1 Pediatric Surgery Division, Federico II University Hospital, Via Pansini 5, 80131 Naples, Italy; 2 Pediatric Surgery Division, Hôpital Necker-Enfants Malades, Paris, France; 3 Pediatric Surgery Division, University Hospital, CHU de Limoges, Limoges, France; 4 Pediatric Surgery Division, Ente Ospedaliero Cantonale (EOC), Bellinzona, Switzerland

**Keywords:** Robot, Gynecology, Tumors, Robotic-assisted, Adolescents, Children

## Abstract

Robotic-assisted surgery (RAS) is increasingly adopted in the pediatric population. This retrospective multicenter study aimed to report application of RAS for gynecological indications in pediatric patients. The medical records of all girls with gynecological pathology, operated in 4 different institutions over a 3-year period, were retrospectively collected. Robot docking time, total operative time, length of stay (LOS), requirement time of pain medication, complication rate, conversion rate, and pathology were analyzed. Twenty-three girls, with median age of 12.3 years (range 0.6–17.8) and median weight of 47.2 kg (range 9–73), received the following RAS procedures: ovarian cystectomy for ovarian cyst/mass (*n* = 10), salpingo-oophorectomy for ovarian complex mass (*n* = 6), bilateral gonadectomy for Turner syndrome SRY + (*n* = 1), salpingectomy for fallopian tube lesion (*n* = 1), paratubal cyst excision (*n* = 1), Gartner cyst excision (*n* = 1), paravaginal ganglioneuroma resection (*n* = 1), fistula closure in urogenital sinus (*n* = 1), and vaginoplasty using ileal flap in cloaca malformation (*n* = 1). Median operative time was 144.9 min (range 64–360), and median docking time was 17.3 min (range 7–50). Conversion to open or laparoscopy was not necessary in any case. Median LOS was 2.1 days (range 1–7), and median analgesic requirement was 2.2 days (range 1–6). One patient (4.3%) needed redo-surgery for recurrent Gartner cyst (Clavien 3b). This preliminary experience showed that RAS is safe and feasible for surgical treatment of gynecological pathology in pediatric patients, although no conclusive data are available to confirm its superiority over traditional laparoscopy. Randomized, prospective, comparative studies are needed to identify the gold standard approach for such indication.

**Supplementary Information:**

The online version contains supplementary material available at 10.1007/s11701-023-01767-9.

## Introduction

Robotic-assisted surgery (RAS) has gained widespread diffusion over the last years, demonstrating the ability to overcome the technical limitations of conventional laparoscopy. Enhancements provided by robotic assistance include three-dimensional view and magnification, increased dexterity with 7-degrees of freedom of robotic instruments, tremor filtering, and improved surgeons’ ergonomics [1, 2]. Some major drawbacks must be considered before using RAS in children: anesthesia, placement of trocars, and technical difficulties related to small space [3]. Nevertheless, RAS has been described as safe and feasible option for a wide range of surgical indications in children, including urological, oncological, and gastrointestinal pathologies [4–8]. Several reports have investigated safety and feasibility of RAS in pediatric population, compared with different approaches (open or laparoscopic) [9–12].

To the current state, the field of pediatric gynecology remains the least explored, with only few pediatric reports of application of RAS for gynecological indications [13–16].

This descriptive, retrospective study aimed to report a multicenter experience regarding the application of RAS for gynecological indications in pediatric patients.

## Materials and methods

All children and adolescents up to 18 years of age, operated using RAS for gynecological indications in 4 different institutions over a 3-year period, were included. The exclusion criteria were patients over 18 years old as well as all gynecological surgical conditions not treated with RAS.

The surgical centers were contacted via mail and those accepting to participate to the study, were required to fill a study form, with requested information, for each enrolled patient.

Patient baseline, including age at time of surgery, weight, possible comorbidities, clinical presentation, pre-operative diagnosis, and side of pathology, were reported in the first section.

Details of operative technique, such as type of procedure, number of robotic and/or accessory ports, use of sealing device, method for specimen extraction, were reported in the second section.

Operative results, including robot docking time, total operative time, length of stay (LOS), requirement time of pain medication, complication rate, conversion rate, pathology, and follow-up results, were analyzed in the third section.

All data were elaborated using the statistical software Microsoft Excel, Windows vers.11. Descriptive statistics were used to present findings, and quantitative variables were expressed as median (range) to report the data.

The study received appropriate Institute Review Board (IRB) approval.

## Results

### Patient baseline

Twenty-three girls, with median age at surgery of 12.3 years (range 0.6–17.8) and median weight of 47.2 kg (range 9–73), received RAS for gynecological indication in the study period and were included. Associated comorbidities were reported in 5/23 (21.7%). Most patients (16/23, 69.5%) were symptomatic at time of diagnosis, with non-specific abdominal/pelvic pain being the most frequent presentation. Pre-operative work-up included abdominal ultrasonography (US), pelvic computed tomography (CT) and/or magnetic resonance imaging (MRI), and voiding cystourethrogram (VCUG) in selected cases. Serum tumor markers, such as beta human chorionic gonadotropin (β-HCG), alfa-fetoprotein (α-FP), Cancer Antigen 125 (Ca125), lactic dehydrogenase (LDH), and human epididymis secretory protein 4 (HE-4), were performed in all patients with adnexal mass. Pre-operative diagnosis was ovarian cyst (*n* = 4), ovarian “complex” mass (*n* = 12), fallopian tube lesion (*n* = 3), uterine cyst (*n* = 1), gonadal dysgenesis (*n* = 1), pelvic paravaginal mass (*n* = 1), cloaca malformation (*n* = 1), and high-confluence urogenital sinus (UGS) (*n* = 1). One patient (4.3%) presented concomitant ovarian “complex” mass and paratubal cyst.

### Installation and operative technique

All RAS procedures were carried out using the da Vinci Xi Surgical System (Intuitive Surgical, Sunnyvale, CA, USA). The patient was placed supine on the operative table and appropriate age-sized Foley catheter was inserted using sterile precautions pre-operatively.

**Fig. 1:**
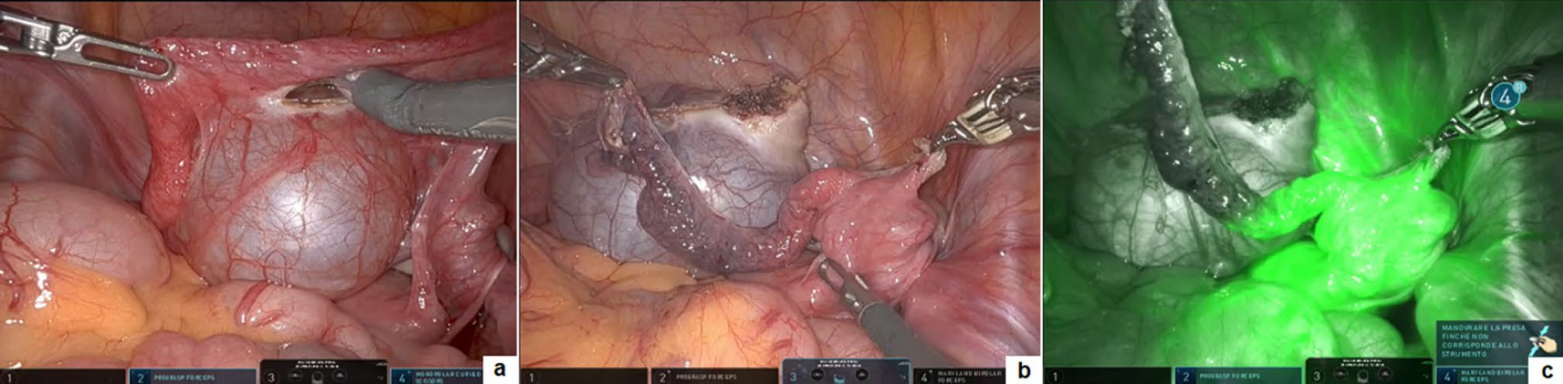
After removal of giant paratubal cyst (**a**, **b**), ICG-guided fluorescence was helpful to check the vascular permeability of fallopian tube (**c**)

Three robotic arms, one 12-mm with 12–8 mm reducer, for the 3D, 0-degree, robotic optic, and two 8-mm ports to accommodate the robotic instruments, were placed on the umbilical line in all procedures. A fourth 5-mm accessory port was also placed. The robot was finally docked over the patient’s feet. Robotic vessel sealer was adopted in all procedures. Indocyanine green (ICG) near-infrared fluorescence (NIRF) was adopted in ovarian mass to check the resection margins and guide intra-operative decision making and in paratubal lesion to check the vascular permeability of the fallopian tube following the removal of the lesion (Fig. 1). A 10-mm bag-retrieval, introduced through the umbilical port, was adopted for specimen extraction.

Video 1 reproduces the technique of robotic-assisted resection of ovarian mass using ICG-NIRF.


Supplementary file1 (MP4 46006KB)

### Operative results

The RAS procedures included: ovarian cystectomy (*n* = 10), salpingo-oophorectomy (*n* = 6), bilateral gonadectomy (*n* = 1), salpingectomy (*n* = 1), paratubal cyst excision (*n* = 1), Gartner cyst excision (*n* = 1), paravaginal ganglioneuroma resection (*n* = 1), fistula closure in UGS (*n* = 1), and vaginoplasty using ileal flap in cloaca malformation (*n* = 1). Median operative time was 144.9 min (range 64–360), and median docking time was 17.3 min (range 7–50). Conversion to open or laparoscopy was not necessary in any case. Median LOS was 2.1 days (range 1–7), and median analgesic requirement was 2.2 days (range 1–6). One patient (4.3%) needed redo-surgery for recurrent Gartner cyst (Clavien 3b).

**Fig. 2:**
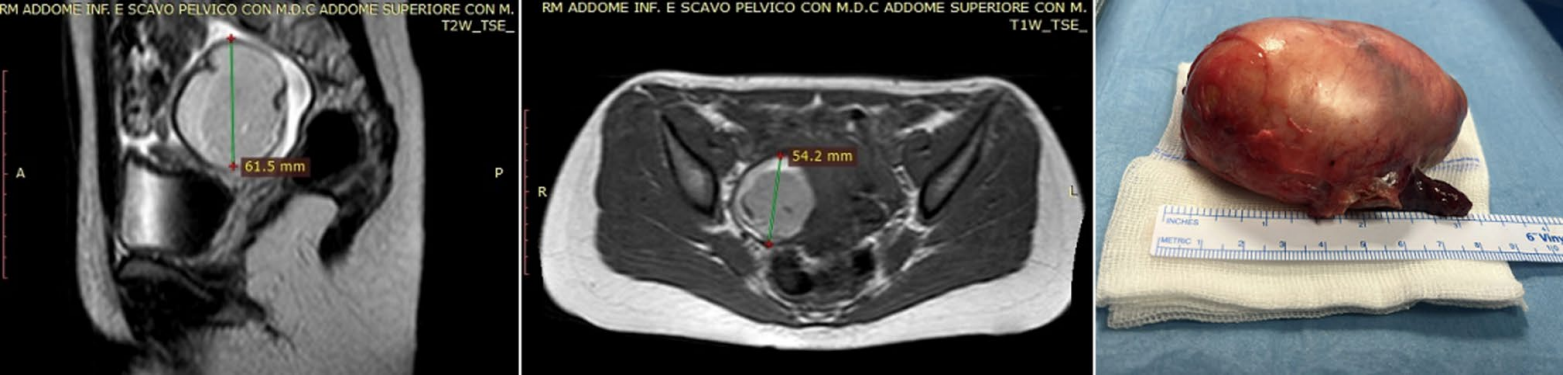
Pre-operative MRI and specimen of right ovarian immature teratoma

The histopathology confirmed diagnosis of ovarian serous cystadenoma (*n* = 2), ovarian functional follicular cyst (*n* = 2), mature cystic teratoma (*n* = 6), immature teratoma (*n* = 4) (Fig. 2), ovotestis (*n* = 2), streak gonads in Turner syndrome SRY + (*n* = 1), paratubal cystadenoma (*n* = 2), Gartner cyst (*n* = 1), and ganglioneuroblastoma (*n* = 1).

The median length of follow-up was 2.2 years (range 0.5–4.5). No patients required adjuvant chemotherapy following surgery and none reported recurrence of tumoral pathology.

All results are summarized in Table 1.

**Table 1:** Outcomes of RAS in pediatric gynecological indications

Patient	Age (years)	Side	Comorbidity	Pre-operative diagnosis	Surgical procedure	OT (min)	LOS (days)	Post-operative complications	Pathology results
1	10	Right	No	Ovarian mass	Salpingo-oophorectomy	85	1	0	Mature cystic teratoma
2	16	Left	No	Giant ovarian cyst (12 cm)	Ovarian cystectomy	72	1	0	Serous cystadenoma
3	17	Right	No	Giant ovarian cyst (10 cm)	Ovarian cystectomy	75	1	0	Serous cystadenoma
4	12	Right	Type I neurofibromatosis	Ovarian mass	Salpingo-oophorectomy	124	3	0	Immature teratoma
5	12	Right	Rheumatoid arthritis	Ovarian mass	Salpingo-oophorectomy	95	2	0	Immature teratoma
6	13	Left	No	Ovarian mass + paratubal cyst	Salpingo-oophorectomy	78	2	0	Immature teratoma + paratubal cyst
7	3	Bilateral	Turner syndrome SRY +	Gonadal dysgenesis	Bilateral gonadectomy	64	2	0	Streak gonads
8	13	Right	No	Fallopian tube lesion	Salpingectomy	96	2	0	Tubal cystadenoma
9	17	N/A	No	Uterine cyst	Excision	200	3	Cyst recurrence (Clavien 3b)	Gartner cyst
10	7	Right	No	Ovarian Mass	Ovarian cystectomy	155	2	0	Mature cystic teratoma
11	14	Left	No	Pelvic paravaginal mass	Excision	198	2	0	Ganglioneuroblastoma
12	10	N/A	No	Cloaca	Vaginoplasty using ileal flap	360	7	0	N/A
13	0.6	N/A	No	Urogenital sinus	Fistula closure	155	3	0	N/A
14	17	Right	DSD	Ovarian mass	Ovarian cystectomy	145	1	0	Ovotestis
15	13	Left	DSD	Ovarian mass	Salpingo-oophorectomy	120	1	0	Ovotestis
16	13	Right	Epilepsy, mild cystic fibrosis	Ovarian mass	Salpingo-oophorectomy	80	1	0	Immature teratoma
17	17.8	Right	Epilepsy, Hashimoto thyroiditis	Paratubal cyst	Paratubal cystectomy	115	2	0	Serous papillary cystadenoma
18	13.2	Left	No	Ovarian mass	Ovarian cystectomy	228	3	0	Mature cystic teratoma
19	12.9	Right	No	Ovarian mass	Ovarian cystectomy	218	2.1	0	Mature cystic teratoma
20	8	Right	No	Ovarian mass	Ovarian cystectomy	185	2.7	0	Mature cystic teratoma
21	14.8	Right	No	Ovarian mass	Ovarian cystectomy	198	2.3	0	Mature cystic teratoma
22	14.7	Bilateral	No	Ovarian cyst (6 cm)	Ovarian cystectomy	155	1.5	0	Functional follicular cyst
23	15	Left	No	Ovarian cyst (7 cm)	Ovarian cystectomy	132	1	0	Functional follicular cyst

*RAS*  robotic-assisted surgery, *OT*  operative time, *LOS* length of stay, *DSD* disorder of sex development

## Discussion

Despite the growing number of indications in pediatric urology, the application of RAS remains still limited in other fields of pediatric surgery. Analyzing the pediatric literature, very few reports on RAS application in gynecology are available, with limited case series or single-case observations [13–15].

Our study collected the number of 4 pediatric surgery units with high volume robotic activity and 24 patients, operated over a 3-year period, were enrolled. Despite the small number of patients included, our preliminary results were promising and showed that RAS may be fully applicable even to gynecological indications in pediatric patients. Improved dexterity, coordination, and visualization were provided by robot assistance. The absence of intra- and post-operative complications also confirmed the safety and feasibility of this approach in children.

Moreover, our study added new elements to the current knowledge. First, the previous reports have described benign ovarian pathology as main indication to RAS [13–15]. Our study introduced further undescribed indications, such as tubal, uterine, and vaginal malformations, and demonstrated the feasibility of complex reconstructive procedures of internal genitalia such as vaginoplasty using ileal flap using robotic approach.

Based on our experience, we believe that the key-points for an optimal management of such pathology using RAS are correct trocar placement, use of sealing device and ICG-NIRF technology, use of endobag for specimen extraction, and teamwork. The most critical step is the placement of trocars, especially in children [17]. Improper placement of the trocars would limit robotic manipulation in the abdominal cavity and increase the chances of instrument conflicts due to the small surface area of the abdominal wall of children and the relatively small space in the abdominal cavity. As described by Xie et al. [14], we always adopted three robotic arms and a fourth accessory laparoscopic port for the bedside surgeon. We placed the robotic ports on the umbilical line to keep proper distance from the pelvic area and have enough working space for manipulation of giant masses or insertion of retrieval bag. Our standard operation order was to insert the trocar for the scope first and then insert the trocars for the robotic arms. Furthermore, if use of specimen retrieval bag is planned, our suggestion is to place 12-mm umbilical robotic port with 12–8 mm reducer to use the 8-mm robotic scope. At time of specimen extraction, the optic is moved to one working arm and the 10-mm retrieval bag is inserted into the abdominal cavity through the umbilical robotic port and the specimen extraction is finally done under direct vision.

Use of robotic vessel sealer is very helpful to perform a bloodless dissection of anatomic structures or resection of giant tumors. In some indications, such as ovarian tumors, use of ICG-NIRF was very helpful to visualize the resection margins of the mass and help guide intra-operative decision between salpingo-oophorectomy and ovarian-sparing surgery. This technology required an intra-operative administration of ICG (0.5 mg/kg) via intravenous route and in a matter of 60 s, fluorescence appeared in the target organs, allowing to identify the resection margins and the vascularization of the mass [18–20]. Recently, ICG-NIRF was also adopted during removal of paratubal lesion, to check the vascular permeability of the fallopian tube following the resection of the lesion.

Use of endobag is needed for extraction of resected tumors with high suspicion of malignancy. We suggest adopting large endobags (volume up to 1000 mL) and extract the specimen by enlarging the umbilical incision and, whenever possible, aspirating the liquid content of cystic masses before extraction, to avoid additional large Pfannestiel incision.

Finally, the teamwork is essential to perform a smooth operation, shorten the learning curve for docking, and ultimately reduce total operative time and anesthetic times.

Limitations of the presented study are the small number series, the limited follow-up period, and the multi-institutional participation, that made the data hardly comparable. The number of accumulated procedures in children is difficult to compare with that in adults. Thus, it was necessary collect the data of different pediatric surgery units to collect more conspicuous evidence.

## Conclusion

This preliminary experience showed that RAS is safe and feasible for surgical treatment of pediatric gynecological pathology, although no conclusive data are available to confirm its superiority over traditional laparoscopy. Case–control and comparative prospective studies will help to delineate better the advantages of this new technology as well as its optimal use in pediatrics. The primary focus for future studies should therefore be on quality management, optimization of patient outcomes for the largest number of patients, and surgical and team training.

## Data Availability

All data will be available on request.
